# Red Blood Cell Distribution Width and Long-Term Outcome in Patients Undergoing Percutaneous Coronary Intervention in the Drug-Eluting Stenting Era: A Two-Year Cohort Study

**DOI:** 10.1371/journal.pone.0094887

**Published:** 2014-04-10

**Authors:** Hai-Mu Yao, Tong-Wen Sun, Xiao-Juan Zhang, De-Liang Shen, You-You Du, You-Dong Wan, Jin-Ying Zhang, Ling Li, Luo-Sha Zhao

**Affiliations:** 1 Department of Cardiology, the First Affiliated Hospital of Zhengzhou University, Zhengzhou, P. R. China; 2 Department of Integrated ICU, the First Affiliated Hospital of Zhengzhou University, Zhengzhou, P. R. China; Washington Hospital Center, United States of America

## Abstract

**Background:**

Previous studies suggest the higher the red blood cell distribution width (RDW) the greater the risk of mortality in patients with coronary artery disease (CAD). However, the relationship between RDW and long-term outcome in CAD patients undergoing percutaneous coronary intervention (PCI) with a drug-eluting stent (DES) remains unclear. This study was designed to evaluate the long-term effect of RDW in patients treated with drug-eluting stent for CAD.

**Methods:**

In total of 2169 non-anemic patients (1468 men, mean age 60.2±10.9 years) with CAD who had undergone successful PCI and had at least one drug-eluting stent were included in this study. Patients were grouped according to their baseline RDW: Quartile 1 (RDW<12.27%), Quartile 2 (12.27%≤RDW<13%), Quartile 3 (13%≤RDW<13.5%), and Quartile 4 (RDW≥13.5).

**Results:**

The incidence of in-hospital mortality and death or myocardial infarction was significantly higher in Quartiles 3 and 4 compared with Quartile 1 (P<0.05). After a follow-up of 29 months, the incidence of all-cause death and stent thrombosis in Quartile 4 was higher than in Quartiles 1, 2, and 3 (P<0.05). The incidence of death/myocardial infarction/stroke and cardiac death in Quartile 4 was higher than in Quartiles 1 and 2 (P<0.05). Multivariate Cox regression analysis showed that RDW was an independent predictor of all-cause death (hazard ratio (HR) = 1.37, 95% confidence interval (CI) = 1.15–1.62, P<0.001) and outcomes of death/myocardial infarction/stroke (HR = 1.21, 95% CI = 1.04–1.39, P = 0.013). The cumulative survival rate of Quartile 4 was lower than that of Quartiles 1, 2, and 3 (P<0.05).

**Conclusion:**

High RDW is an independent predictor of long-term adverse clinical outcomes in non-anemic patients with CAD treated with DES.

## Introduction

Red blood cell distribution width (RDW) is an objective measure of the heterogeneity in red blood cell (RBC) size (i.e., it is a coefficient of variability of RBC volume). RDW is obtained from RBC size distribution, and is commonly utilized in the differential diagnosis of anemia. A number of studies report that high levels of RDW are associated with increased mortality among patients with heart failure, myocardial infarction (MI), or coronary artery disease (CAD), and in those undergoing percutaneous coronary intervention (PCI) [1]–[6]. A high RDW is also associated with elevated cardiovascular biomarkers and cardiac enzymes [7]. Study of a Chinese population showed that elevated RDW predicts an increased risk of short-term adverse outcomes in patients with acute coronary syndrome [8].

The initial success rate of PCI is high; therefore, long-term follow-up results are most appropriate for evaluating the predictive value of RDW. However, the long-term prognostic value of RDW in patients with anemia is uncertain [6], [9], since many studies did not exclude patients in the general population with anemia, thus affecting outcome. Previous studies of the prognostic value of RDW in patients undergoing PCI included those treated with drug-eluting stent (DES) or bare metal stent (BMS). While the clinical endpoints were the same regardless of choice of stent, DES was associated with a significantly reduced incidence of in-stent restenosis and need for revascularization of the target lesion. However, the potential for thrombosis following DES is a concern.

For the reasons outlined above we conducted a prospective observational cohort study to investigate the prognostic value of RDW in patients treated with DES.

## Methods

### Ethics Statement

The study was approved by the Ethics Committee of the first affiliated hospital of Zhengzhou University. All aspects of the study comply with the Declaration of Helsinki. Ethics Committee of the first affiliated hospital of Zhengzhou University specially approved that not informed consent was required because data were going to be analyzed anonymously.

### Study Population

This study recruited consecutive patients without anemia who underwent PCI from July 2009 to August 2011 at a single large-volume PCI center. Qualitative and quantitative coronary angiographic analyses were carried out according to standard methods. PCI was performed using standard techniques. All patients were given loading doses of aspirin (300 mg) and clopidogrel (300 mg) before the coronary intervention, unless they had already received these antiplatelet medications. The treatment strategy, stenting techniques, selection of stent type, as well as use of glycoprotein IIb/IIIa receptor inhibitors or intravascular ultrasound were all left to the operator's discretion. Daily aspirin (100 mg) and clopidogrel (75 mg) were prescribed for at least the first 12 months after the procedure. Patients were excluded from analysis if they had been referred for urgent PCI following acute MI, if they had a history of blood transfusion or if they presented with cardiogenic shock

### Definitions used in the study

Cardiovascular risk factors were assessed at the time of hospital admission. Patients ≥65 years old were defined as being elderly. A history of smoking was assumed if the patient had smoked within the last10 years. Patients were classed as having diabetes mellitus if their fasting plasma glucose concentration was >6.1 mmol/L, their hemoglobin A1c (HbA1c) was >6.5%, or if they were currently being treated with insulin or oral hypoglycemic agents. Patients were defined as having hypertension if their systolic blood pressure was ≥140 mmHg, their diastolic blood pressure was ≥90 mmHg, or if antihypertensive drugs were prescribed. Dyslipidemia was defined as low-density lipoprotein cholesterol >140 mg/dL, high-density lipoprotein <40 mg/dL, or lipid-lowering drugs were prescribed. Anemia was defined as a hemoglobin level <12.0 g/dL in women and <13.0 g/dL in men, based on the World Health Organization definition [10]. Glomerular filtration rate was estimated by the Cockcroft-Gault formula [11]. Target vessel revascularization (TVR) was defined as a repeat procedure, either PCI or coronary artery bypass grafting (CABG) in the target vessel. Stent thrombosis (ST) was proven by angiography, or assumed as probable if an unexplained sudden death occurred within 30 days after stent implantation, or if a Q-wave MI was diagnosed in the distribution area of the stented artery. This classification was issued according to definitions proposed by the Academic Research Consortium (ARC): acute (<24 h), subacute (24 h to 30 days), late (1–12 months), and very late (>12 months) [12]. Major bleeding was defined using REPLACE-2 criteria [13], which include decreases in hematocrit of ≥12%, and in hemoglobin of ≥4 g/dL, transfusion of ≥2 units of packed RBC and retroperitoneal, gastrointestinal or intracranial bleeding.

### Classification of RDW

Patients were divided into four groups according to their baseline RDW: Quartile 1 (RDW<12.27%), Quartile 2 (12.27%≤RDW<13%), Quartile 3 (13%≤RDW<13.5%), and Quartile 4 (RDW≥13.5%).

### Clinical Outcomes and data collection

Prospective data were entered into a database that contained demographic, clinical, angiographic, and procedural information. Primary endpoints included all-cause death, occurrence of MI, stent thrombosis, and TVR. The composite endpoint was defined as MACCE, namely death/MI/stroke. Clinical follow-up was carried out through patient visits, telephone interview, and medical record review. Data were entered by independent research personnel and clinical events were adjudicated by physicians who were not involved in the procedures themselves. All deaths were considered to be cardiac unless an unequivocal noncardiac cause could be established. Cardiac deaths included all events related to a cardiac diagnosis, any complication of a procedure and treatment thereof, or any unexplained cause. Unexpected death, even in patients with a coexisting and potentially fatal noncardiac disease (e.g., cancer or infection), was classified as cardiac unless their history relating to the noncardiac diagnosis suggested death was imminent. Between July 2009 and August 2011, 2348 non-anemic patients in our hospital were treated with at least one DES. Data were collected from 2169 patients (92.4%) over a follow-up period of 29.1±5.3 months (range 25.5–33.2months).

### Statistics

Continuous variables were expressed as mean ± standard deviation (SD). Categorical variables were expressed as percentages. Normally distributed continuous variables were analyzed using one-way ANOVA test. Variables whose distribution could not be assumed to be normal were analyzed using the nonparametric Wilcoxon Rank-Sum tests. The Chi-square or Fisher's Exact test were used for categorical variables. Cumulative survival curves were constructed using the Kaplan–Meier method and comparisons made using log-rank tests. Cox regression analysis was performed to identify independent predictors of death and MACCE. All baseline, demographic, clinical, and angiographic variables were entered into the model. Results were reported as hazard ratios (HRs) and 95% confidence intervals (CIs). All statistical tests were 2-tailed, and p values were statistically significant at <0.05. All data were analyzed with SPSS 17.0 (SPSS Inc., Chicago, Illinois, USA)

## Results

### Baseline clinical characteristics

Demographic characteristics of the 2169 patients of the study are shown in Table 1. The mean age was 60.2±10.9 years and 67.7% of the patients were men. RDW ranged from 9.3% to 23% (mean, 12.9±1.25%).

**Table 1:** Baseline characteristics of participants by Quartile of RDW.

	Quartile 1	Quartile 2	Quartile 3	Quartile 4	P value
	(n = 539)	(n = 578)	(n = 546)	(n = 506)	
RDW	11.36±0.65	12.65±0.23	13.23±0.18	14.39±1.13	0.000
Age, year	58.3±11.0	58.6±10.7	60.1±10.6	63.2±10.8	<0.001
Age>65 years	170(31.5)	181(31.3)	210(38.5)	243(48)	0.000
Male gender	383(71.1)	393(68)	377(69)	315(62.3)	0.018
BMI, kg/m^2^	23.5±5.3	23.8±6.5	24.0±7.8	23.6±5.0	0.79
Prior PCI	40(7.4)	29(5.0)	42(7.7)	36(7.1)	0.32
Prior CABG	3(0.6)	7(1.2)	4(0.7)	4(0.8)	0.62
Prior myocardial infarction	35(6.5)	41(7.1)	64(11.7)	78(15.4)	0.000
Peripheral vessel disease	1(0.2)	1(0.2)	0(0)	2(0.4)	0.52
COPD	2(0.4)	7(1.2)	3(0.5)	7(1.4)	0.19
Heart Failure	42(7.8)	52(9.0)	43(7.9)	52(10.3)	0.29
Prior stroke	20(3.7)	26(4.5)	38(7.0)	38(7.5)	0.016
Risk factors					
Arterial hypertension	208(38.6)	224(38.8)	314(57.5)	348(68.8)	0.000
Dyslipidemia	294(54.5)	323(55.9)	299(54.8)	272(53.8)	0.92
Diabetes mellitus	89(16.5)	122(21.1)	125(22.9)	157(31)	0.000
Current smoker	175(32.5)	175(30.3)	202(37)	152(30)	0.052
GFR, ml·min^−1^·1.73 m^−2^	73.9±14.6	73.4±14.8	71.3±15.6	68.7±16.1	<0.001
Clinical presentation					
Stable angina	31(6.5)	32(5.5)	31(5.7)	38(7.5)	0.53
Unstable angina	355(65.9)	387(67)	378(69.2)	331(65.4)	0.55
Non-STEMI	8(1.5)	6(1.0)	6(1.1)	4(0.8)	0.76
STMI	128(23.7)	122(21.1)	110(20.1)	98(19.4)	0.32
Total cholesterol, mmol/L	4.24±1.01	4.24±1.06	4.31±1.05	4.31±1.14	0.56
Triglyceride, mmol/L	1.90±1.36	1.97±1.15	1.92±1.40	1.84±1.55	0.53
LDL-C, mmol/L	2.68±0.93	2.67±0.93	2.7±0.90	2.7±0.97	0.93
HDL-C, mmol/L	1.06±0.34	1.03±0.29	1.07±0.30	1.11±0.32	0.002
Glycemia, mmol/L	5.82±2.36	6.24±3.69	5.86±2.18	6.13±4.11	0.09
Haemoglobin, g/L	141.6±11.2	141.3±9.6	141.2±12.3	140.7±12.6	0.23
Medication at discharge					
Aspirin,	537(99.6)	568(98.3)	537(98.4)	499(98.6)	0.17
Clopidogrel	537(99.6)	576(99.7)	545(99.8)	505(99.8)	0.91
ACEI/ARB	286(53.1)	325(56.2)	297(54.4)	286(56.5)	0.63
Beta-blocker	380(70.5)	419(72.5)	397(72.7)	341(67.4)	0.20
Statins	507(94.1)	554(95.8)	511(93.6)	473(93.5)	0.28

Values are mean ± SD or n (%). RDW: red blood cell distribution width, PCI: percutaneous coronary intervention, CABG: coronary artery bypass graft, COPD: chronic obstructive pulmonary disease, GFR: glomerular filtration rate, STEMI: ST segment elevation myocardial infarction, Non-STEMI: Non-ST segment elevation myocardial infarction, LDL-C: Low density lipidprotein cholesterol, HDL-C: High density lipidprotein cholesterol, ACEI: Angiotensin-converting enzyme inhibitor, ARB: angiotensin recptor blocker.

There were no significant differences among the groups with respect to body mass index, clinical presentation, medications at discharge, or hemoglobin level. The percentages of patients with prior revascularization, heart failure, or peripheral vessel disease, and the percentage of current smokers were similar in all four groups. However, patients with higher RDW values tended to be older and to have more cardiovascular risk factors and more cardiovascular diseases (prior MI and prior stroke) compared with patients with lower RDW values. Other factors that were independently associated with baseline RDW level are shown in Table 1.

Angiographic and procedural characteristic

The proportion of patients with a type B1 lesion was lower in Quartiles 3 and 4 than in Quartile 1 (p<0.001). Compared with quartile 1, the proportion of patients with a type B2 or type C lesion was significantly higher in quartile 4 (p<0.05). The left ventricular ejection fraction (LVEF) was significantly lower in quartile 4 than in Quartiles 1, 2, and 3 (p<0.05). By contrast, the location of the target lesion, the numbers of vessels treated and stents inserted, total stent length and stent diameter were not significantly different among the groups (Table 2).

**Table 2:** Baseline angiographic and procedural characteristics of participants by Quartile of RDW.

	Quartile 1	Quartile 2	Quartile 3	Quartile 4	P value
	(n = 539)	(n = 578)	(n = 546)	(n = 506)	
Radial artery access^a^	528(98)	567(98.3)	532(97.4)	490(96.8)	0.35
Number of diseased vessels^a^					
1-vessel disease	222(41.2)	233(40.3)	203(37.2)	183(36.2)	0.29
2-vessel disease	196(36.4)	208(36)	214(39.2)	191(37.7)	0.69
3-vessel disease	120(22.3)	132(22.8)	129(23.2)	133(26.3)	0.44
Location of lesion^a^					
Left main stem	14(2.8)	16(2.8)	21(3.8)	18(3.6)	0.59
LAD	442(82)	485(83.9)	444(81.3)	408(80.6)	0.52
LCX	255(47.3)	265(45.8)	265(48.5)	269(53.2)	0.097
RCA	253(46.9)	273(47.2)	277(50.7)	256(50.6)	0.43
LVEF^b^, (n = 1470)	61.2±7.5	61.2±7.1	61.4±6.3	59.4±8.6	0.005
LVEF<40%	3(0.6)	9(1.6)	8(1.5)	15(3.0)	0.021
Type of target lesion according to AHA/ACC^a^ (n = 3426)					
A	83(10.1)	73(8.7)	75(8.5)	74(8.3)	0.58
B1	303(36.9)	276(33)	251(28.5)	227(25.6)	0.000
B2	241(29.3)	270(32.3)	311(35.3)	322(36.3)	0.01
C	195(23.5)	217(26)	244(27.7)	264(29.8)	0.035
Total chronic occlusion	40(7.4)	55(9.5)	47(8.4)	59(11.7)	0.11
Ostial lesions	53(9.8)	62(10.7)	57(10.4)	43(8.5)	0.63
Number of treated vessels^b^	1.51±0.67	1.5±0.65	1.53±0.65	1.51±0.66	0.92
Location of target lesions^a^					
Left main stem	16(3.0)	12(2.1)	17(3.1)	12(2.4)	0.67
LAD	389(72.2)	424(73.4)	390(71.4)	345(68.2)	0.28
LCX	209(38.8)	207(35.8)	203(37.2)	205(40.5)	0.42
RCA	201(37.3)	234(40.5)	223(40.8)	210(41.5)	0.51
NO. of stents per patient^b^	2.14±1.33	2.12±1.22	2.13±1.24	2.21±1.26	0.74
Total stent length per patient^b^	50.0±34.2	49.5±32.1	49.7±32.5	51.2±32.8	0.89
Stent Diameter (mm)^b^	3.01±0.44	3.12±0.43	3.06±0.43	3.04±0.45	0.57

RDW: red blood cell distribution width, LVEF: Left ventricular ejection fraction, LAD: left anterior descending artery, LCX: left circumflex artery, RCA: right coronary artery.

a : n(%),

b : mean ± SD.

### Clinical Outcomes

The incidence of in-hospital death and of MACE (death/MI) was significantly higher in quartile 4 than in quartiles 1 and 2 (p<0.05), but the incidence of any MI did not vary among the groups (p>0.05).

During the mean follow-up of period 29 months, higher baseline levels of RDW were associated with an increased risk of all-cause death, cardiac death, MACCE, and stent thrombosis. When participants were grouped according their baseline RDW quartile the graded relationship between RDW and all-cause death, cardiac death and stent thrombosis remained. The incidence of cardiac death and MACCE was significantly higher in quartile 4 than in quartiles 1 and 2 (p<0.005). The incidence of all-cause death and stent thrombosis in quartile 4 was significantly higher than in quartiles 1, 2, and 3 (p<0.001). There were no significant differences among the groups with respect to the incidence of nonfatal MI, nonfatal stroke, any revascularization (PCI/CABG), or in-stent restenosis (Table 3).

**Table 3:** Clinical events from PCI until discharge and end of follow up by Quartile of RDW.

	Quartile 1	Quartile 2	Quartile 3	Quartile 4	P value
	(n = 539)	(n = 578)	(n = 546)	(n = 506)	
In-hospital events, n (%)					
Death	1(0.2)	2(0.35)	5(0.9)	8(1.6)	0.047
Any MI	4(0.7)	2(0.35)	5(0.9)	5(1.0)	0.61
MACE(death or MI)	5(0.9)	4(0.7)	10(1.8)	13(2.6)	0.049
Follow-up(cumulated events), n (%)					
All cause death	11(2.0)	12(2.1)	14(2.6)	29(5.7)	0.001
Cardiac death	6(1.1)	9(1.6)	12(2.2)	19(3.8)	0.02
Nonfatal MI	6(1.1)	5(0.9)	9(1.6)	11(2.2)	0.31
Nonfatal stroke	6(1.1)	4(0.7)	11(2.0)	9 (1.8)	0.26
MACCE(death/MI/stroke)	21(3.9)	22(3.8)	30(5.5)	42(8.3)	0.004
Major bleeding	4(0.7)	3(0.5)	3(0.5)	6(1.2)	0.57
Any revascularization(PCI/CABG)	46(8.5)	42(7.3)	41(7.5)	41(8.1)	0.86
TVR	24(4.5)	21(3.6)	27(5.0)	22(4.3)	0.71
In-stent restenosis	35(6.5)	29(5.0)	35(6.4)	27 (5.3)	0.64
Stent thrombosis(definite/probable)	7(1.3)	8(1.4)	13(2.4)	27(5.3)	0.00

RDW: red blood cell distribution width, MACE: major adverse cardiac event, MI: myocardial infarction, PCI: percutaneous coronary intervention, CABG: coronary artery bypass graft, MACCE: major cardiovascular or cerebral adverse events, TVR: target vessel revascularization.

### Univariate and multivariate analysis

**Figure 1:**
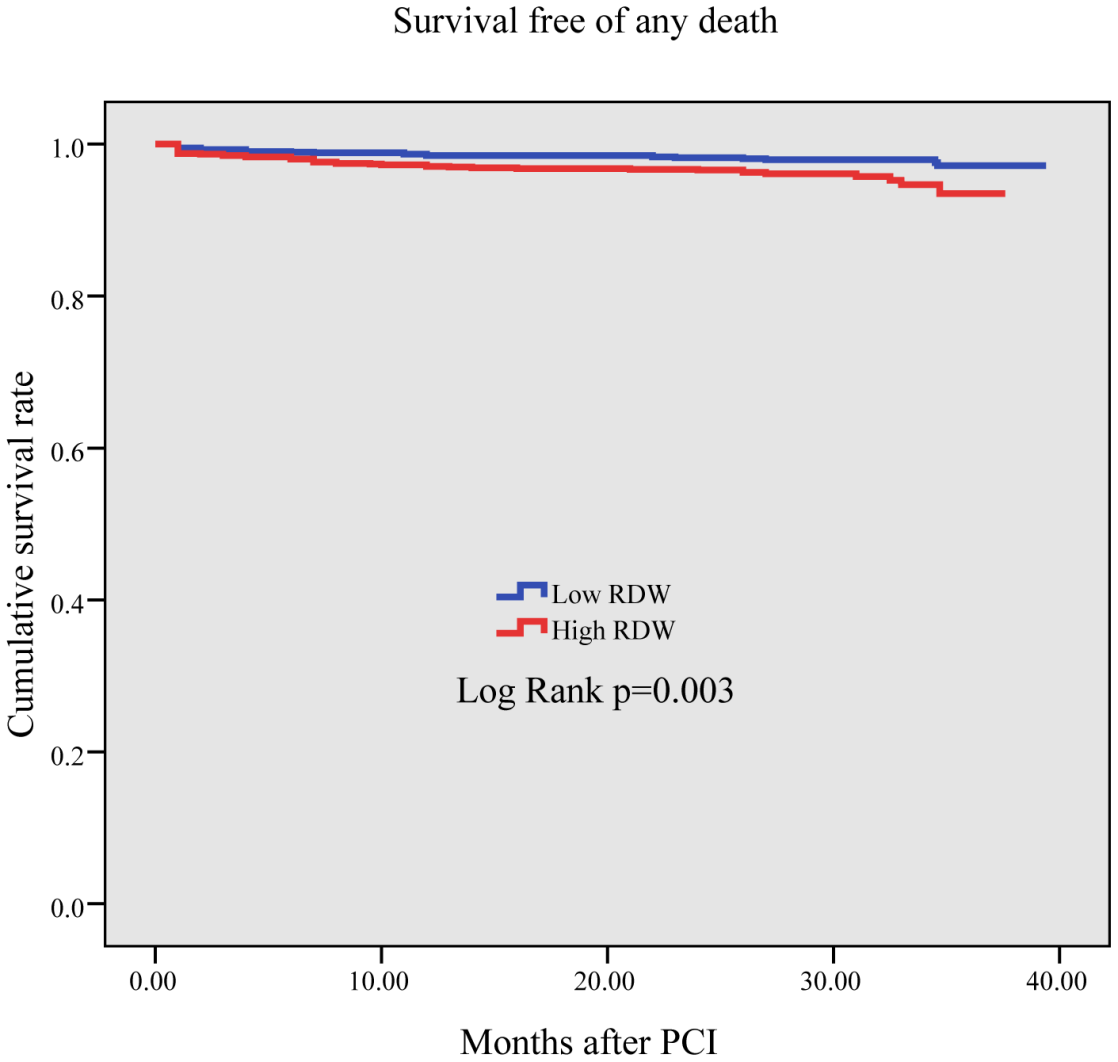
The Kaplan–Meier curve of all-cause mortality rate. It is significantly higher in quartiles 2, 3, and 4 than in quartile 1 (P<0.001).

**Figure 2:**
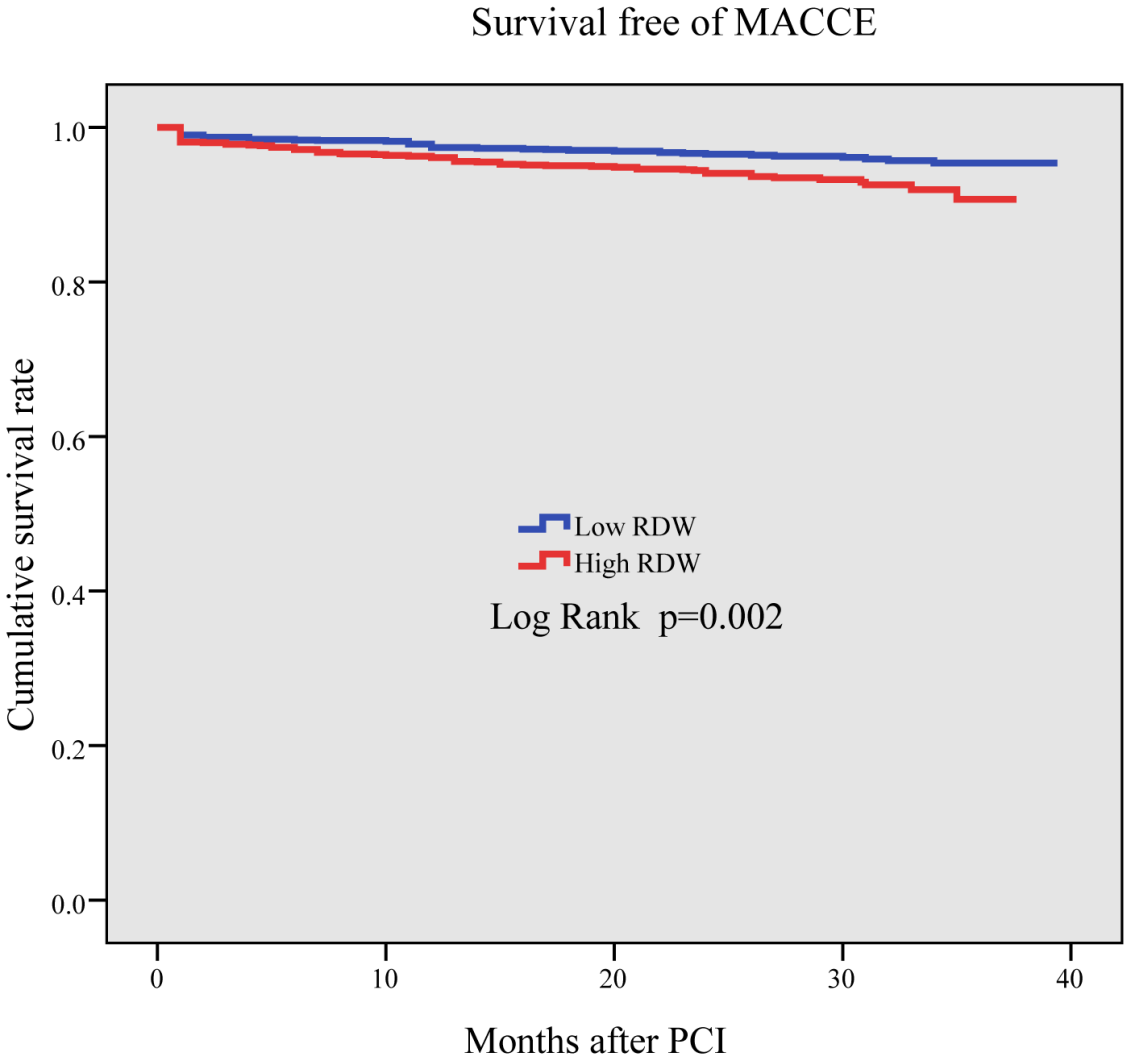
The Kaplan–Meier curve of MACCE rate. It is significantly higher in quartiles 3 and 4 than in quartiles 1 and 2 (p = 0.002).

Results of univariate analyses for all-cause death are shown in Table 4, and for MACCE are shown in table 5. Multivariate Cox regression analysis was used to assess the predictors of all-cause death and MACCE. After adjusting for age, gender, diabetes mellitus, hypertension, peripheral vascular disease, number of vessels treated, multi-vessel disease, prior MI, GFR, LVEF, number of stents implanted, total stent length, and stent diameter, the continuous variable RDW was significantly associated with both an increased incidence of all-cause death (HR = 1.37, 95% CI = 1.15–1.62, P<0.001) and MACCE (HR = 1.21, 95% CI = 1.04–1.39, P = 0.013) (Table 6). The Kaplan–Meier curve revealed that the cumulative survival rate in quartiles 2, 3, and 4 was lower than in quartile1 (P<0.001, Figure 1) and the cumulative survival rate free from MACCE in quartiles 3 and 4 was lower than in quartiles 1 and 2 (p = 0.002, Figure 2)

**Table 4:** Univariate Analysis for All-Cause death.

	HR	95%CI	P value
RDW (per 1%)	1.4	1.23–1.59	0.000
Age(years)	1.07	1.04–1.09	0.000
Gender(male)	0.857	0.52–1.41	0.55
Hypertension	1.07	0.66–1.72	0.79
Dyslipidemia	0.89	0.51–1.56	0.68
Total cholesterol	0.82	0.64–1.10	0.13
HDL-C	0.64	0.26–1.56	0.33
LDL-C	0.93	0.71–1.23	0.63
Triglyceride	0.82	0.63–1.08	0.16
Heart failure	0.96	0.45–2	0.9
Diabetes mellitus	1.8	1.08–3.0	0.024
Peripheral vascular disease	8.1	1.1–58	0.038
Prior myocardial infarction	1.88	0.99–3.59	0.055
Cerebral vascular disease	1.3	0.55–3.38	0.51
Number of treated vessels	1.16	0.89–1.60	0.371
number of stents implanted	1.16	0.97–1.48	0.11
total stent length	1.01	1.001–1.015	0.031
the stent diameter	0.72	0.41–1.26	0.69
STEMI	1.26	0.73–2.2	0.41
history of revascularization	2.38	0.68–11.4	0.22
GFR (ml·min^−1^·1.73 m^−2^)	0.93	0.86–0.98	0.013
Multi-vessel disease	2.97	1.84–4.79	0.000
LVEF≤40%	3.09	2.11–4.52	0.000
DAPT	0.41	0.18–0.95	0.038
Use of ACEI/ARB	1.01	0.62–1.62	0.97
Use of β-blockers	0.66	0.40–1.07	0.96
Use of statins	0.75	0.30–1.86	0.53
Use of insulin	1	0.37–2.76	0.99

RDW: Red blood cell distribution width, HR: hazard ratio, CI: confidence interval, HDL-C: High density lipidprotein cholesterol, LDL-C: Low density lipidprotein cholesterol, STEMI: ST-segment elevation myocardial infarction, GFR: glomerular filtration rate, LVEF: Left ventricular ejection fraction, DAPT: dual antiplatelet therapy, ACEI: Angiotensin-converting enzyme inhibitor, ARB: angiotensin recptor blocker.

**Table 5:** Univariate Analysis for MACCE.

	HR	95%CI	P value
RDW (per 1%)	1.27	1.13–1.42	0.000
Age(years)	1.056	1.04–1.08	0.000
Gender(male)	1.10	0.74–1.63	0.65
Hypertension	1.17	0.81–1.69	0.401
Dyslipidemia	1.034	0.66–1.62	0.88
Total cholesterol	0.97	0.81–1.16	0.75
HDL-C	0.83	0.44–1.56	0.56
LDL-C	1.034	0.85–1.26	0.74
Triglyceride	1.03	0.0.91–1.16	0.69
Heart failure	0.75	0.403–1.4	0.37
Diabetes mellitus	1.43	1.02–2.85	0.044
Peripheral vascular disease	5.01	0.699–35.86	0.075
Prior myocardial infarction	1.46	0.85–2.51	0.17
Cerebral vascular disease	0.913	0.401–2.08	0.83
Number of treated vessels	1.28	1.06–1.56	0.013
number of stents implanted	1.13	0.99–1.29	0.07
total stent length	1.01	1.001–1.011	0.25
the stent diameter	0.45	0.28–0.73	0.001
STEMI	1.204	0.79–1.84	0.39
history of revascularization	1.58	0.64–7.4	0.35
GFR(ml·min^−1^·1.73 m^−2^)	0.94	0.81–0.99	0.021
Multi-vessel disease	2.29	1.58–3.33	0.000
LVEF≤40%	1.92	1.35–2.74	0.000
DAPT	0.54	0.21–0.86	0.035
Use of ACE-I/ARB	0.975	0.68–1.41	0.89
Use of β-blockers	0.644	0.44–0.94	0.021
Use of statins	0.57	0.31–1.06	0.076
Use of insulin	0.87	0.38–1.98	0.74

RDW: Red blood cell distribution width, HR: hazard ratio, CI: confidence interval, HDL-C: High density lipidprotein cholesterol, LDL-C: Low density lipidprotein cholesterol, STEMI: St-segment elevation myocardial infarction, GFR: glomerular filtration rate, LVEF: Left ventricular ejection fraction, DAPT: dual antiplatelet therapy, ACEI: Angiotensin-converting enzyme inhibitor, ARB: angiotensin recptor blocker.

**Table 6:** Multivariate analysis for All-Cause Mortality and MACCE.

	HR	95% CI	P value
All-Cause death			
RDW (per 1%)	1.37	1.15–1.62	0.000
Age(years)	1.064	1.03–1.1	0.000
GFR ml·min^−1^·1.73 m^−2^)	0.91	0.78–0.96	0.037
Multi-vessel disease	2.15	1.19–3.85	0.011
LVEF≤40%	2.89	1.96–4.25	0.000
MACCE			
RDW (per 1%)	1.21	1.04–1.39	0.013
Age(years)	1.053	1.03–1.08	0.000
GFR(ml·min^−1^·1.73 m^−2^)	0.94	0.86–0.98	0.005
LVEF≤40%	1.58	1.08–2.31	0.02

RDW: Red blood cell distribution width, HR: hazard ratio, CI: confidence interval, MACCE: Major cardiovascular or cerebral adverse events, GFR: glomerular filtration rate, LVEF: Left ventricular ejection fraction.

## Discussion

Variability in the size of circulating red cells (anisocytosis) is reflected by RDW, which is routinely reported by automated laboratory equipment for complete blood counts. Although its use has been limited to narrowing the differential diagnosis of anemia, mounting evidence suggests that there are additional roles for this measurement.

The present study demonstrates that high RDW is an independent marker of a long-term adverse prognosis in non-anemic patients with CAD treated with DES. We found this association to be independent of multiple potential confounding factors, including age, renal insufficiency, diabetes mellitus, peripheral vascular disease, prior MI, and left ventricular dysfunction. Additionally, a higher RDW is also associated with a higher incidence of both cardiac death and stent thrombosis (definite/probable) during follow-up. The results suggest that RDW may be used to stratify patients treated with DES according to long term-risk. Therefore, particularly as no additional cost is incurred, closer evaluation of RDW is recommended.

Poludasu et al. [4] reported that RDW is an independent predictor of mortality in patients undergoing PCI; however, the association was only observed between RDW and mortality and their sample size was relatively small. In addition, their study included just those patients who had undergone bolus-only treatment with glycoprotein IIb/IIIa antagonists and excluded patients who had ST-segment elevation MI, some of whom may have been high risk, thus reducing the study's ability to demonstrate any relationship. Fatemi et al. [6] demonstrated the additive value of RDW in a larger cohort of patients undergoing coronary angiography. The cohort did not include consecutive patients and follow-up was limited to 1 year. Recently, in a larger cohort study, Arbel et al. [14] investigated the prognostic value of RDW in patients after PCI. They excluded patients with acute heart failure and did not analyze the association between RDW and in-hospital events. In addition, they did not collect data about bleeding events or stent thrombosis. Furthermore, all three studies included patients treated with either DES or BMS, and grouped anemic and non-anemic patients together in the analysis. Our study demonstrates the importance of RDW for both in-hospital and long-term clinical outcome in a large Chinese population treated with DES. Many studies have reported that the use of DES is associated with improved prognosis after PCI [15], [16], and DES is widely used in current clinical practice (over 98% of patients were treated with DES at our center). While DES and BMS share the same “hard” clinical endpoints, DES is associated with a significant reduction in both the incidence of in-stent restenosis and the need for revascularization of the target lesion. However, there is a concern over stent thrombosis after DES insertion, such that it is necessary to evaluate the prognostic value of RDW in patients treated with DES. Our data confirm and extend the prognostic significance of an elevated RDW in patients with heart failure [17], in patients post-MI without heart failure [1], and in those with chest pain referred to coronary angiography [5].

Anemia and hemoglobin levels are strong predictors for the development of CVD and mortality in a variety of populations [18]–[19]. A community cohort study [9] revealed that an elevated RDW is significantly associated with all-cause mortality in people without anemia, but that the mortality risks for patients with anemia are the same regardless of RDW. Furthermore, the association between high RDW and death not attributable to CVD was only significant in subjects without anemia. Recently, in a large community-based cohort study, Arbel et al. [20] found that, over a 5-year follow-up, elevated RDW levels were significantly associated with increased risk of cardiovascular morbidity and all-cause mortality in patients with and without anemia, the association being stronger in the latter group. The reasons for the differences between these studies were unclear; therefore, to exclude the effect of anemia on clinical outcome, our study only enrolled those patients without anemia. Our results show that elevated RDW is significantly associated with not only all-cause mortality and cardiac death but also the incidence of MACCE.

An interesting finding in our study was that higher RDW is also associated with an increased incidence of stent thrombosis, but not with major bleeding. Fatenmi et al. [21] reported that elevated RDW is an independent predictor of bleeding after PCI. In a study of 3845 adult outpatients, RDW was shown to have an independent, graded relationship with high-sensitivity C-reactive protein (CRP) and erythrocyte sediment rate [22]. The relationship between inflammation, (which predisposes to thrombosis) and bleeding risk is a paradox whose cause may lie in the complex pathology of patients in poor health [23]. Frail, elderly patients have relatively higher levels of CRP, factor VIII, and D-dimer [24] and increased levels of activated factors VII, IX, and X [25], leading to an increased likelihood of thrombosis. Arbel et al. [14] reported that elevated RDW is associated with abnormal bone marrow function, which itself is related to increased platelet activation, aggregation and thrombus activation [26]. Further studies are required to confirm the association between RDW and major bleeding and to explore its underlying mechanisms of action.

It remains unclear how elevated RDW is associated with adverse cardiovascular outcomes. Chronic subclinical inflammation is the pathway most frequently hypothesized, as it is a well-established entity preceding de novo cardiovascular events and can adversely influence erythropoiesis by a variety of mechanisms, including direct myelosuppression of erythroid precursors, reduced renal erythropoietin production and iron bioavailability, increased erythropoietin resistance in erythroid precursor cell lines, the promotion of cell apoptosis, and RBC membrane deformability, all factors that might increase anisocytosis [27]. Recently, in a large cohort study included of 8513 adults participants (age>20 years) with no pre-existing cardiovascular disease, Veeranna et al. [28] found that RDW but not hs-CRP is associated with mortality from coronary heart disease. This suggests that mechanisms other than inflammation may be involved, for example oxidative stress, tissue hypoxia or endothelial dysfunction [29]–[30]. Furthermore, higher RDW may reflect enhanced erythropoiesis resulting from elevated circulating levels of neurohumoral mediators [2]. There is increasing evidence that the existence of a chronic inflammatory state and neurohumoral activation both contribute to adverse clinical outcomes in patients with acute coronary syndrome [5], [31], [32]. Additionally, an elevated RDW is often seen in patients with extensive comorbidities and therefore correlates with increased morbidity and mortality.

Our study also has some limitations that should be considered. First, this was a post-hoc observational analysis, and therefore we cannot rule out the possibility of residual confounding. However, the hypothesis that RDW levels are associated with adverse outcomes was formulated before analyses began, reducing the risk of drawing spurious conclusions. Second, this was a single-center study. Patients who were referred for urgent PCI or who were in cardiogenic shock were excluded from this study, because their incidence of adverse outcomes is known to be high; therefore, there might be selection bias. Baseline characteristics across the RDW groups were reasonably consistent but adjustments had to be made for certain differences, in particular the increased prevalence of comorbidity in patients with a high RDW. Despite the adjustment for multiple variables, it is possible that residual unrecognized confounding variables influenced the observed differences in outcome between the groups. As with all analyses of observational data, this study cannot distinguish causality from association. Third, factors such as iron, vitamin B12, and folate were not measured in this study. Finally, because hs-CRP was not measured in most of patients, we were unable to analyze the relationship between RDW and hs-CRP.

### Conclusion

In conclusion, our study demonstrates that elevated RDW predicts an increased risk of adverse clinical outcomes, both in hospital and over the long-term, in non-anemic patients with CAD treated with DES, and that RDW has the potential for use as a tool for risk stratification. Further studies are required to confirm the association between RDW and clinical outcomes and to elucidate its underlying mechanism.
