# Correction: Red Blood Cell Distribution Width and Long-Term Outcome in Patients Undergoing Percutaneous Coronary Intervention in the Drug-Eluting Stenting Era: A Two-Year Cohort Study

**DOI:** 10.1371/journal.pone.0103461

**Published:** 2014-07-18

**Authors:** The PLOS ONE Staff

Figures 1 and 2 are incorrect.

Please see the correct Figure 1 here.

**Figure 1:**
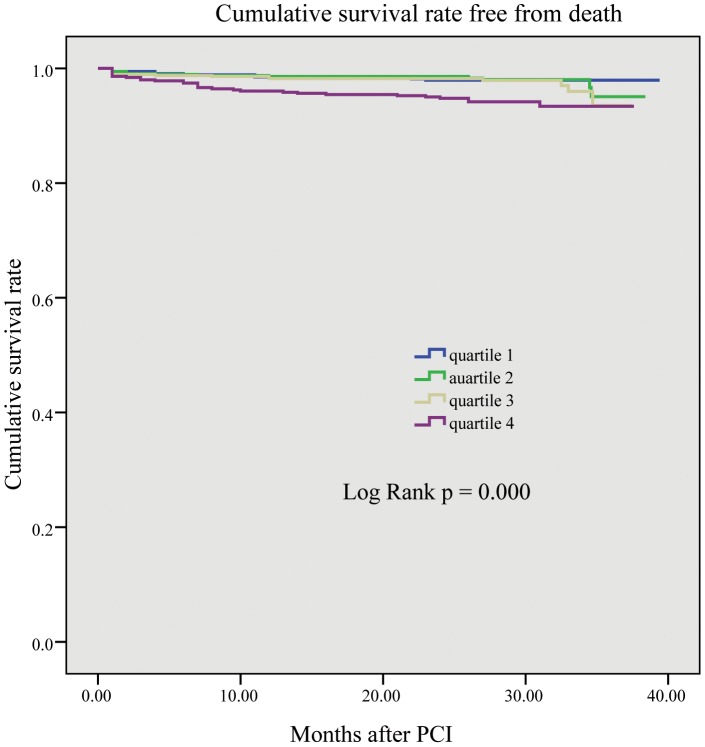
The Kaplan–Meier curve of all-cause mortality rate. It is significantly higher in quartiles 2, 3, and 4 than in quartile 1 (P<0.001).

Please see the correct Figure 2 here.

**Figure 2:**
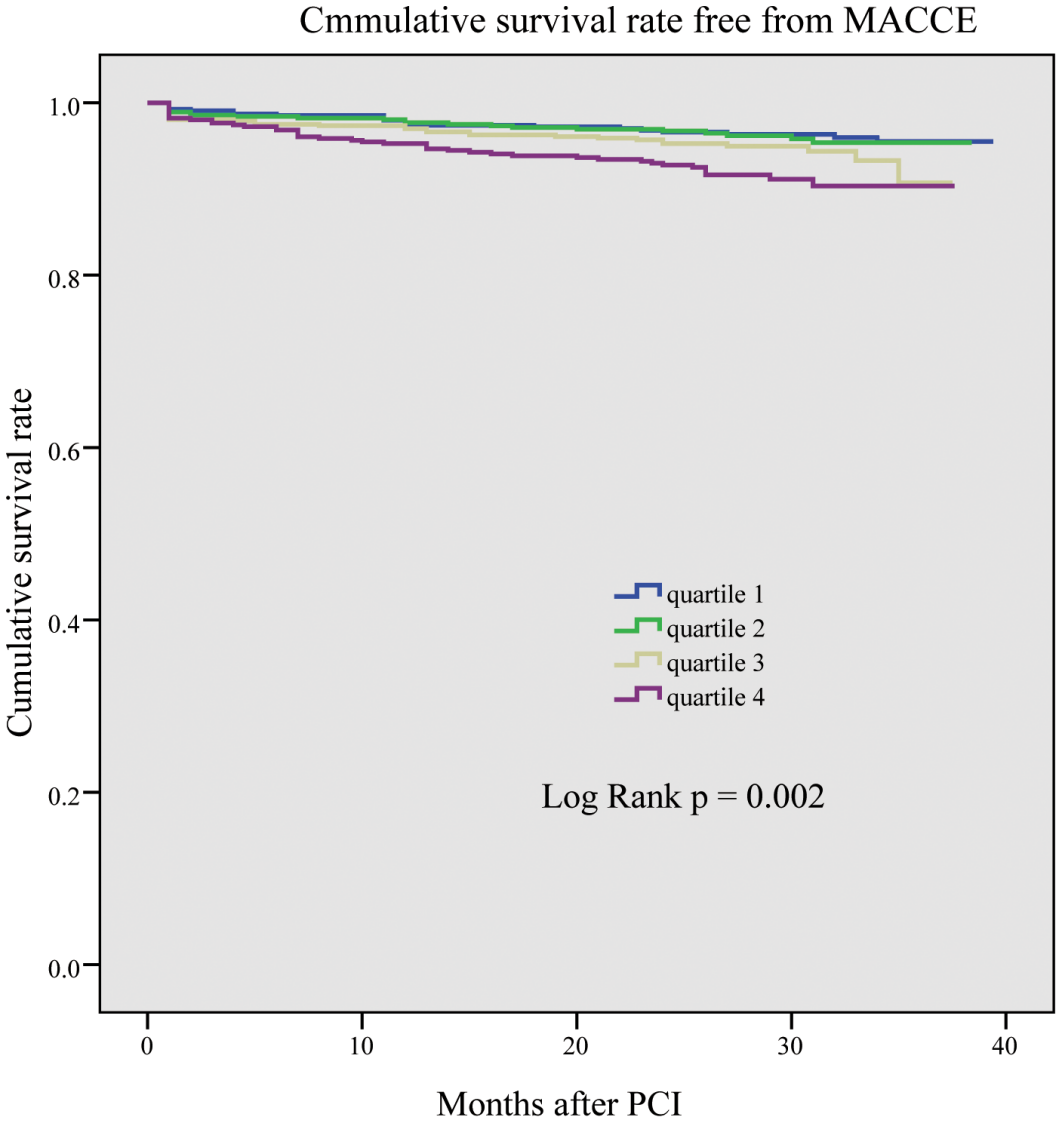
The Kaplan–Meier curve of MACCE rate. It is significantly higher in quartiles 3 and 4 than in quartiles 1 and 2 (p  =  0.002).
